# Physicochemical Properties and Chemical Stability of β-Carotene Bilayer Emulsion Coated with Bovine Serum Albumin and Arabic Gum Compared to Monolayer Emulsions

**DOI:** 10.3390/molecules23020495

**Published:** 2018-02-23

**Authors:** Bulei Sheng, Lin Li, Xia Zhang, Wenjuan Jiao, Di Zhao, Xue Wang, Liting Wan, Bing Li, Hui Rong

**Affiliations:** 1 College of Food Science and Engineering, South China University of Technology, 381 Wushan Road, Guangzhou 510640, China; shengbulei@outlook.com (B.S.); felinli@scut.edu.cn (L.L.); wjiao.scut@hotmail.com (W.J.); di.zhao.scut@hotmail.com (D.Z.); wangxue12344321@126.com (X.W.); nbwlting@163.com (L.W.); 2 Guangdong Province Key Laboratory for Green Processing of Natural Products and Product Safety, 381 Wushan Road, Guangzhou 510640, China; 3 School of Chemical Engineering and Energy Technology, Dongguan University of Technology, College Road 1, Dongguan 523808, China; 4 Guangzhou Entry-Exit Inspection & Quarantine Bureau of China, Guangzhou 510623, China; rongh@gdciq.gov.cn

**Keywords:** β-carotene, bilayer emulsion, bovine serum albumin (BSA), Arabic gum (GA), physicochemical property, chemical stability

## Abstract

β-carotene is a lipophilic micronutrient that is considered beneficial to human health. However, there are some limitations in utilizing β-carotene in functional foods or dietary supplements currently because of its poor water dispersibility and chemical stability. A new type of β-carotene bilayer emulsion delivery system was prepared by a layer-by-layer electrostatic deposition technique, for which were chosen bovine serum albumin (BSA) as the inner emulsifier and Arabic gum (GA) as the outer emulsifier. The physicochemical properties of bilayer emulsions were mainly characterized by droplet size distribution, zeta potential, rheological behavior, Creaming Index (CI), and encapsulation ratio of β-carotene. Besides this, the effects of processing conditions (pH, thermal treatment, UV radiation, strong oxidant) and storage time on the chemical stability of bilayer emulsions were also evaluated. The bilayer emulsion had a small droplet size (221.27 ± 5.17 nm) and distribution (PDI = 0.23 ± 0.02), strong zeta potential (−30.37 ± 0.71 mV), good rheological behavior (with the highest viscosity that could reduce the possibility of flocculation) and physical stability (CI = 0), high β-carotene encapsulation ratio (94.35 ± 0.71%), and low interfacial tension (40.81 ± 0.86 mN/m). It also obtained better chemical stability under different environmental stresses when compared with monolayer emulsions studied, because it had a dense and thick bilayer structure.

## 1. Introduction

β-Carotene is a carotenoid that functions as a colorant and antioxidant in foods. It can be found in many vegetables and fruits, including carrots, pumpkins, tomatoes, broccoli, and mangoes [1]. β-carotene is also the precursor of vitamin A (retinol). A molecule of β-carotene can be converted to two molecules of vitamin A in vivo by β-carotene 15,15’-monoxygenase with the highest transformation activity among all carotenoids [2,3,4]. Therefore, an appropriate amount of β-carotene can improve our vision to decrease myopia [5] and is beneficial to embryo growth [6] by cleavage into vitamin A. Besides this, proper intake of β-carotene can also enhance human’s antioxidant capacity [7], reduce the risk of type 2 diabetes [8] and cardiovascular disease [9,10], alleviate metabolic syndrome in middle-aged people [11], and strengthen the immune system [9]. However, the β-carotene within natural food is difficult to dissolve in water and easy to degrade [10], resulting in low bioavailability [12,13]. Therefore, to enhance the bioavailability of β-carotene, it is important to prepare a stable and efficient delivery system to enhance its water dispersibility and chemical stability [14].

A delivery system based on oil-in-water (o/w) emulsion can be employed to improve the water dispersibility of β-carotene to increase its bioavailability [15,16] because β-carotene has good solubility in edible oil [17]. Traditionally, o/w emulsions are prepared using a single emulsifier (small molecule, protein, or polysaccharide) or compound emulsifier (composed of two or more emulsifiers) [18], in which the oil droplets are surrounded by a single interfacial layer. The monolayer emulsions are susceptible to environmental stresses such as heat, light, pH, oxygen, and free radicals, thus limiting in their applications in the food or medicine industry [19].

A bilayer emulsion prepared by a layer-by-layer electrostatic deposition technique (LBL) [20,21,22] is an alternative and promising strategy in protecting and delivering liposoluble bioactive components. A bilayer emulsion is produced when an emulsifier or a polyelectrolyte is added to an already-formed monolayer emulsion and adsorbed on the droplet surfaces under certain pH [23]. The thick membrane coat outside the droplets can improve the chemical stability of the emulsions [24]. Nowadays, bilayer emulsion delivery systems comprising lecithin and chitosan [25], β-lactoglobulin and pectin [26,27,28], sodium caseinate and carboxymethyl cellulose [29], sodium caseinate and xanthan gum [30], α-actalbumin and chitosan–EGCG conjugates [31], whey protein isolate (WPI) and oxidized starch adhesive [32], whey separation protein and beet pectin [33,34], and soybean soluble polysaccharide and chitosan [35,36] have been reported. Among these systems, the droplet size distribution of the monolayer emulsion prepared using WPI was large and the membrane was thin because WPI is a mixture of globular proteins [37,38]. The membrane made with casein was porous with different thicknesses, since casein is a mixture of liner flexible proteins [37,38,39]. β-lactoglobulin and α-actalbumin are expensive in cost; moreover, β-lactoglobulin can cause allergies [40]. Chitosan is not easy to dissolve in water and smells bad [41]; worse, chitosan would reduce the digestibility of oil and the bioavailability of carotenoids [20]. Therefore, emulsifiers/polyelectrolytes involved in establishing a bilayer emulsion system should be carefully chosen. They should have some specific properties, such as low cost, a double-layer-forming capacity, ease of operation, and no adverse effects on the properties of bilayer emulsions.

The objective of the current study was to design a new type of bilayer emulsion delivery system with good physicochemical properties, comprising bovine serum albumin (BSA) as the inner emulsifier and Arabic gum (GA) as the outer emulsifier. As a globular protein, BSA would form a thin but dense membrane on the oil droplet with a small emulsion droplet size [42]. GA is widely used in the food industry [21,43,44]. These two kinds of emulsifiers both have good water solubility and are cheap, which facilitates industrial application. Compared with the monolayer emulsion, the physicochemical properties of the bilayer emulsion were investigated, and their chemical stability under different environmental stresses was evaluated.

## 2. Results and Discussion

### 2.1. Physicochemical Properties of the Monolayer and Bilayer Emulsions

Droplet size, size distribution, and zeta potential are very important parameters of emulsion droplets. Small droplets contribute to establishing a thermodynamically stable system and efficiently enhance the water dispersibility [45,46]. A small size distribution means that all droplets have almost the same size without too much difference; this indicates a good quality of emulsion in which partial flocculation will seldom occur. Besides this, when emulsion droplets have a strong zeta potential greater than 30 mV, the emulsion will obtain a good physical stability; this is because strong electrostatic repulsion hinders the contact of emulsion droplets with each other, which prevents the aggregation of droplets and reduces the possibility of flocculation in emulsion [21]. Table 1 presents the *z*-average diameter, polydispersity index (PDI), and zeta potential of the monolayer and bilayer emulsions. As shown in Table 1, GA-e had the biggest droplet size and PDI (*p* < 0.05), because GA is a weak emulsifier with a linear stereo structure and large molecular weight. As a result, GA was difficult to anchor to the oil droplet and the thickness of the GA membrane varied, which caused the size distribution to become bigger than those of the other two emulsions (*p* < 0.05) [45]. BSA is a globular protein which can easily form a thin membrane to surround the oil droplet with almost the same thickness [44], so BSA-e had the smallest droplet size and PDI. The *z*-average diameter of BSA/GA-e was significantly bigger (*p* < 0.05) than that of BSA-e and its PDI only increased a little, which indicates that the structure of the bilayer emulsion is uniform without partial flocculation. All three emulsions had strong zeta potentials at pH 4. BSA-e had a positive zeta potential, while GA-e and BSA/GA-e had negative zeta potentials. BSA-e with its positive zeta potential could absorb negative GA to further form the bilayer emulsion whose electric property was then determined by the outer layer [21]. Droplet properties can be derived from the characteristics of emulsifiers or polyelectrolytes themselves but also from the preparation conditions. Too much emulsifier involved would cause bridging flocculation, while too little emulsifier would cause depletion flocculation [45]. In comparison with monolayer emulsions, bilayer emulsions are more sensitive to the added concentration of the second emulsifier/polyelectrolyte [21,47]. Therefore, when preparing a bilayer emulsion, it is important to remove the excess first emulsifier before adding the next emulsifier/polyelectrolyte so that the rate of flocculation is reduced [22].

The β-carotene encapsulation ratio is another important parameter in industrial production. Too much β-carotene could not dissolve in the oil in the pre-experiments. Therefore, the concentration of β-carotene in the oil phase was 0.1%—smaller than that in other emulsions [31]. As shown in Table 1, GA-e had the lowest β-carotene encapsulation ratio (*p* < 0.05), because the GA membrane was reticulate with lots of slits [48,49] so that oil can permeate through the membrane and form an oil layer upon the emulsion. BSA-e had the highest ratio because BSA is a strong emulsifier and can be adsorbed to the oil droplet rapidly to form a dense membrane [37]. As a result of the second homogenization, the β-carotene encapsulation ratio of BSA/GA-e was not significantly different than that of BSA-e (*p* > 0.05). Table 1 indicates that the interfacial tension of GA-e was largest (*p* < 0.05) because GA is a weaker emulsifier than BSA. BSA/GA-e had a small value of interfacial tension because the droplets of it were stabilized by two kinds of emulsifiers together to form a more stable bilayer structure [50].

### 2.2. Rheological Behavior of the Monolayer and Bilayer Emulsions

**Figure 1:**
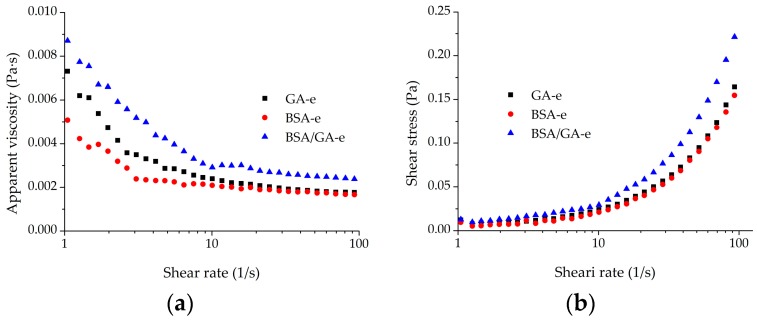
The variation of apparent viscosity (**a**) and shear stress (**b**) with the shear rate of the three emulsions studied.

The rheological behavior is another significant property of food emulsions ranging from highly non-Newtonian viscoelastic products to mobile Newtonian liquids. Careful manipulation of emulsion rheology is generally essential to preparing emulsion products with good textures [51]. As shown in Figure 1, the apparent viscosity and shear stress of the monolayer and bilayer emulsions are illustrated as a function of frequency. In all cases, Figure 1a,b depict that the apparent viscosity of all emulsions decreased along with the rise of the shear rate, while the shear stress increased as the shear rate increased. Flow curves (Figure 1b) were expressed using the Herschel–Bulkley model, and Table 2 shows the corresponding parameters. The high correlation coefficient R^2^ (>0.9) shows that the Herschel–Bulkley model fits the flow curves very well. The consistency index *κ* represents the viscous nature of the emulsion, and higher *κ* reflects higher viscosity [36]. In this study, the *κ* of bilayer emulsions was higher (*p* < 0.05) than that of monolayer emulsions, which suggests the improved viscosity of emulsions. The viscosity increase of the bilayer emulsions may be attributed to the network between BSA and GA formed by electrical interactions between droplets [52]. The flow behavior *n* < 1 indicates a shear-thinning fluid and *n* = 1 represents a Newtonian fluid [53]. Both monolayer and bilayer emulsions displayed shear-thinning behavior, which means that GA did not change the fluid behavior of BSA-e. For a typical shear-thinning emulsion, pseudoplasticity is associated with disruption behaviors of floc structures at high shear rate [54,55]. Therefore, both monolayer and bilayer emulsions may exhibit the same breaking down behaviors of flocculated droplets with the increase of shear rate. The results were similar to those of other bilayer emulsions formed by a-lactalbumin/sodium caseinate and chitosan-(−)-epigallocatechin-3-gallate [31].

### 2.3. Evaluation of the Chemical Stability of the Monolayer and Bilayer Emulsions

#### 2.3.1. pH

**Figure 2:**
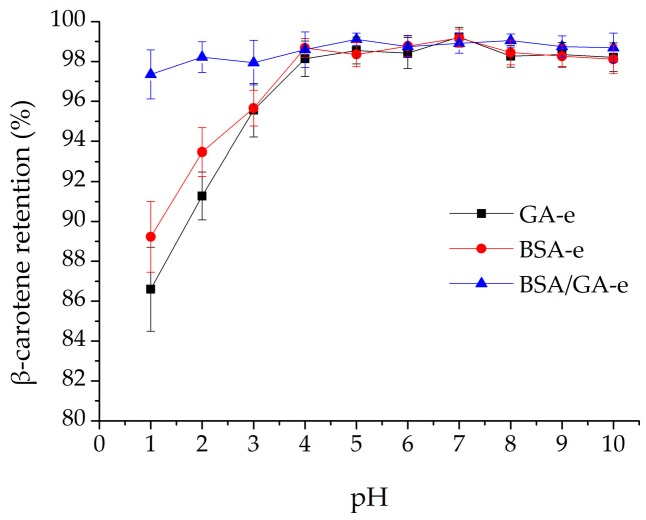
The effects of pH on the β-carotene retention of the three emulsions studied. The pH of the three emulsions studied were adjusted to 1, 2, 3, 4, 5, 6, 7, 8, 9, 10 separately and they were stored at room temperature without light for 2 h. Then β-carotene retention was measured after storage. Each data point is an average of triplicate, and the standard deviations are given.

β-Carotene is sensitive to pH, especially under low-pH conditions, because it may degrade [10]. Therefore, the delivery system is required to have the ability to protect the β-carotene at low pH. As depicted in Figure 2, the bilayer emulsion BSA/GA-e could reduce the degradation of β-carotene efficiently from pH 1 to 10. However, monolayer emulsions GA-e and BSA-e lost more β-carotene than did BSA/GA-e when the pH was below 4, especially for GA-e (*p* < 0.05, data not shown). BSA/GA-e had a dense and thick bilayer membrane which resisted the influence of low pH on the chemical stability of β-carotene in BSA/GA-e. The membrane structure of GA-e was reticulate with lots of slits [48,49], so that the oil phase could contact the low-pH environment easily. As a globular protein, BSA built a thin membrane which may form some cracks when protein denaturalizes in the low-pH conditions [56].

#### 2.3.2. Thermal Treatment

**Figure 3:**
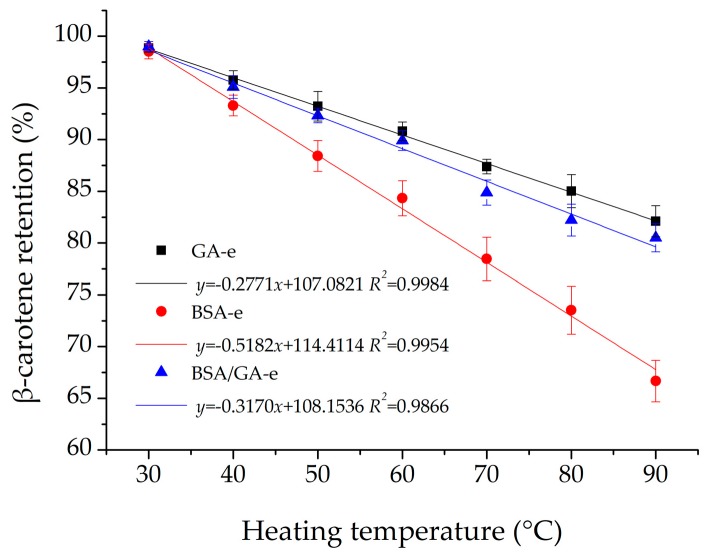
The effects of heating temperature on the β-carotene retention of the three emulsions studied. The temperature of the three emulsions studied were adjusted to 30 °C, 40 °C, 50 °C, 60 °C, 70 °C, 80 °C, 90 °C separately and they were stored without light for 2 h. β-carotene retention was then measured after storage. Each data point is an average of triplicate, and the standard deviations are given. Solid lines represent the fitting results from the linear equation using OriginPro.

Thermal treatment is often applied during the actual processing of food, such as pasteurization (normally 60~82 °C). β-carotene degrades easily or changes into its cis-isomer when the temperature is too high, especially in emulsions [10]. Figure 3 indicates that the results of the three emulsions studied all fitted the linear equation and BSA-e had the smallest slope (−0.5182), while the slopes of GA-e and BSA/GA-e were −0.2771 and −0.3710, respectively. Therefore, the β-carotene in BSA-e degraded much faster than that in GA-e or BSA/GA-e with the increase of temperature. This phenomenon might be explained by the fact that the membrane formed by BSA was thin and the droplet size of BSA-e was small, which means it had larger surface area. As a result, the heating area of BSA-e was expansive and the thin membrane did not have good heat insulation. Besides this, thermal treatment also caused unfolding of BSA to form a thinner membrane [57]. Droplets in GA-e had a thick membrane and big size with limited heating area [58], so that it could weaken the influence of heating, thus protecting the β-carotene. Although the droplet of BSA/GA-e was small, it had a thicker membrane than did the BSA-e emulsions, which helped to reduce the degradation of the β-carotene efficiently. Besides this, thermal treatment caused flocculation in BSA/GA-e to decrease the heating area. Bilayer emulsions formed by lactoferrin and lactoferrin-(−)-epigallocatechin-3-gallate/lactoferrin–chlorogenic conjugates had similar results [59].

#### 2.3.3. UV Radiation

**Figure 4:**
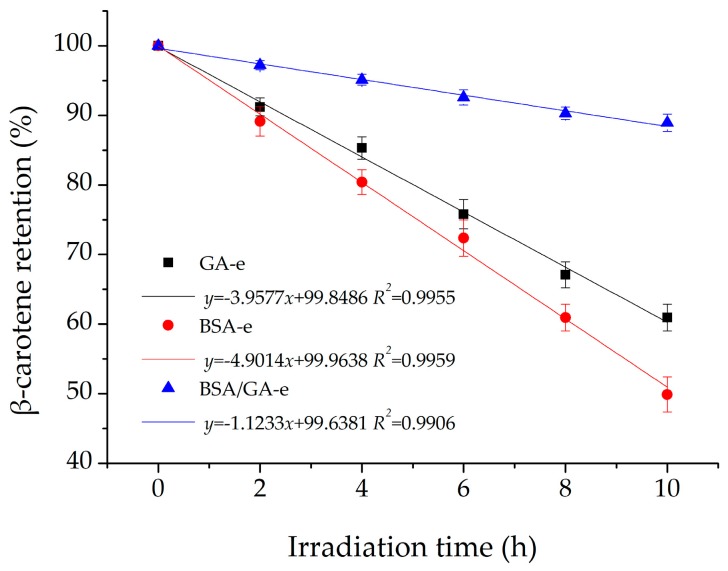
The effects of UV irradiation on the β-carotene retention of the three emulsions studied. The β-carotene retention of the three emulsions studied were measured after the UV radiation for 0, 2, 4, 6, 8, 10 h. Each data point is an average of triplicate, and the standard deviations are given. Solid lines represent the fitting results from the linear equation using OriginPro.

β-Carotene becomes unstable under illumination and easily degrades [10]. Accordingly, it is essential for the emulsion delivery system to reduce the effect of light. This study investigated the influence of UV radiation on the chemical stability of β-carotene in three emulsions. As shown in Figure 4, the results of the three emulsions studied all fitted the linear equation and BSA/GA-e had the biggest slope (−1.1233), while the slopes of GA-e and BSA-e were −3.9577 and −4.9014, respectively. Therefore, BSA/GA-e lost much less β-carotene than did GA-e or BSA-e with the radiation time increasing. This could be attributed to their different membrane structures. Some beams could pass through the slits in the membrane of GA-e, and the ability of BSA-e to protect β-carotene from radiation was limited because of its thin membrane. However, droplets in BSA/GA-e were coated by the dense and thick bilayer membrane which could block and weaken radiation at the same time. In addition, the UV light treatment did not change the *z*-average diameter, PDI, or zeta potential of droplets in all emulsions (data not shown).

#### 2.3.4. Strong Oxidant

**Figure 5:**
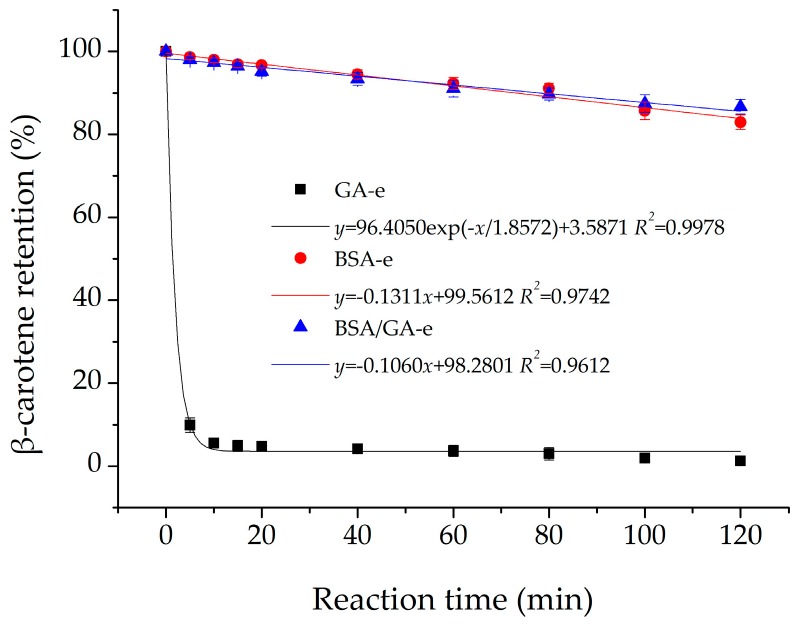
The effects of NaClO on the β-carotene retention of the three emulsions studied. The β-carotene retention of the three emulsions studied were measured after the reaction for 0, 5, 10, 15, 20, 40, 60, 80, 100, 120 min. Each data point is an average of triplicate, and the standard deviations are given. Solid lines represent the fitting results from the linear (BSA-e and BSA/GA-e) and exponential (GA-e) equation using OriginPro.

In some liquid environments, such as tap water, there are usually some metal ions or ionic groups with strong oxidizing ability, such as Fe^3+^ and ClO^−^, which could degrade β-carotene rapidly [10]. Therefore, an emulsion delivery system should prevent contact between β-carotene and strong oxidants in aqueous phase as far as possible. In this study, pure NaClO was added into each emulsion at room temperature to study the effect of strong oxidant on the stability of β-carotene. Figure 5 shows that the results of BSA-e and BSA/GA-e fitted the linear equation and their slopes were small with little difference. However, the results of GA-e fitted the exponential equation. Therefore, NaClO caused quick degradation of β-carotene in GA-e, while BSA-e (slope = −0.1311) and BSA/GA-e (slope = −0.1060) could maintain β-carotene in high quantity even when as the reaction time increased. The slits in the membrane of droplets in GA-e would increase the possibilities for NaClO to come into contact with the β-carotene. Although BSA-e had a thin membrane, it was dense so as to protect the β-carotene against strong oxidants in aqueous phase. BSA/GA-e contained an inner layer of BSA, providing the same protection for the β-carotene. Additionally, the thicker membranes of BSA/GA-e might hinder the interactions between β-carotene and prooxidants in the aqueous phase, thus decreasing β-carotene degradation rates [60].

#### 2.3.5. Storage Time

**Figure 6:**
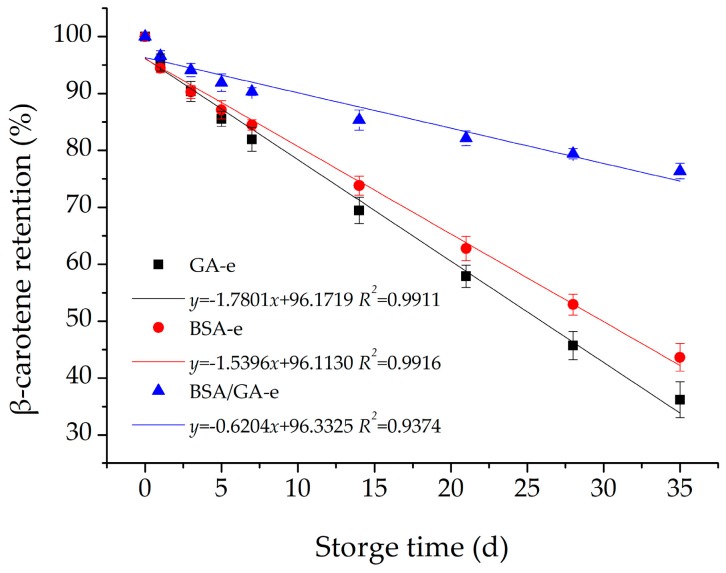
The effects of storage time (25 °C) on the β-carotene retention of the three emulsions studied. The β-carotene retention of the three emulsions studied were measured after storage without light for 0, 1, 3, 5, 7, 14, 21, 28, 35 d. Each data point is an average of triplicate, and the standard deviations are given. Solid lines represent the fitting results from the linear equation using OriginPro.

The storage time of β-carotene delivery system would determine the shelf life of its end products. The monolayer and bilayer emulsions were stored under the same conditions (25 °C refrigerator without light) for five weeks. Figure 6 depicts that the results of the three emulsions studied all fitted the linear equation and BSA/GA-e had the biggest slope (−0.6204), while the slopes of GA-e and BSA-e were −1.7801 and −1.5396, respectively. Therefore, BSA/GA-e had the least loss of β-carotene and a slowed speed of degradation. However, GA-e and BSA-e did not increase the chemical stability of β-carotene efficiently during storage. This could be explained by former experiment results that GA-e had some slits in its membrane, which reduced its ability to protect β-carotene from low pH, light, and oxidants in aqueous phase and caused β-carotene to permeate through the membrane. On the other hand, BSA could only build a single-layer membrane, so that the β-carotene in BSA-e could not be kept stable in a low-pH, high-temperature, or illuminated environment. However, BSA/GA-e had a good chemical stability for β-carotene in different conditions because of its dense and thick bilayer structure. These results are similar to some extent to those of laccase-treated emulsions coated by proteins and sugar beet pectin, because the latter emulsions have crosslinked interface layers made up of ferulic acid, pectin, and proteins [61,62]. Previous studies have reported that the enzymatic crosslinking of polymer layers can improve storage stability and pH stability of contents in emulsions [61,62]. Besides this, their droplet size, size distribution, and zeta potential did not change remarkably, especially for BSA/GA-e (data not shown).

## 3. Materials and Methods

### 3.1. Materials

β-Carotene with purity ≥93% and sodium azide were purchased from Sigma-Aldrich Corp. (St. Louis, MO, USA). Bovine serum albumin (BSA) was purchased from BioFroxx Corp. (Pfungstadt, Germany). Arabic gum (GA) was purchased from Lingfeng Chemical Reagents Co., Ltd. (Shanghai, China). Medium-chain triglyceride (MCT) oil was purchased from Yuanye Biotech Co., Ltd. (Shanghai, China). NaCl and NaOH were purchased from Damao Chemical Reagents Factory (Tianjin, China). NaClO, acetic acid and sodium acetate were purchased from Qiangsheng Functional Chemical Co., Ltd. (Suzhou, China). Concentrated hydrochloric acid, ethanol, and hexane were purchased from National Drug Group Chemical Reagents Co., Ltd. (Beijing, China). All other chemicals were of analytical grade, unless otherwise stated.

### 3.2. Preparation of β-Carotene Emulsions

#### 3.2.1. Preparation of Aqueous and Oil Phase

Aqueous A (BSA solution) and B (GA solution) were prepared by respectively adding BSA (1.5 wt %) and GA (1.5 wt %) into deionized water and stirring (1000 r/min) for 30 min to ensure complete dispersion and dissolution. Sodium azide (0.02 wt %) was added as an antimicrobial agent to prevent microbial growth during sample preparation. The oil phases of the emulsions were prepared by adding β-carotene (0.1 wt %) into MCT and stirring (500 r/min) for 10 min to ensure complete dispersion and dissolution.

#### 3.2.2. Monolayer Emulsion Preparation

The optimal protein or polysaccharide content required to prepare stable monolayer emulsions was investigated in preliminary experiments. BSA monolayer emulsion (BSA-e) and GA monolayer emulsion (GA-e) were prepared by mixing aqueous A or B, respectively, with 2.5 wt % oil phase at a speed of 10,000 r/min for 3 min using an Ultra-Turrax T18 (IKA-Werke GMBH & CO., Staufen, Germany). The coarse emulsions were subsequently homogenized using a Panda Plus two-stage valve homogenizer (GEA Corp., Düsseldorf, Germany) at the operational pressure of 70 MPa as the first stage pressure and 7 MPa as the second stage pressure for three cycles.

#### 3.2.3. Bilayer Emulsion Preparation

BSA/GA bilayer emulsion (BSA/GA-e) was prepared by mixing BSA-e (25 wt %) and aqueous B (75 wt %), followed by adjusting the pH to 4 with 1 M HCl. The system was then homogenized by three passes at 70 MP as the first stage pressure and 7 MPa as the second stage pressure through a two-stage valve homogenizer. As a control, the pH of monolayer emulsions was adjusted to 4.0 and they were then diluted with an equal volume of pH-adjusted deionized water (pH 4.0) to make the final oil concentration the same as that of the bilayer emulsion [47].

### 3.3. Measurements of Droplet Size and Size Distribution

The droplet size and size distribution of emulsions were measured by utilizing a Zetasizer Nano-ZSP (Malvern Instruments Ltd., Worcestershire, UK) at a fixed angle of 90°. The emulsions were diluted 100 times with 0.01 M acetate buffer (pH 4.0) to minimize multiple scattering effects. The droplet size and size distribution were described using the *z*-average diameter and polydispersity index (PDI), respectively.

### 3.4. Measurements of Zeta Potential

The zeta potential of the oil droplets in the emulsions was determined by measuring the direction and velocity of droplet movement in the applied electric field with the aid of a Zetasizer Nano-ZSP (Malvern Instruments Ltd., Worcestershire, UK). All emulsions were diluted 100 times with 0.01 M acetate buffer (pH 4.0) prior to zeta potential analysis.

### 3.5. Measurement of Interfacial Tension of Emulsions

The interfacial tension of emulsions was determined by the hanging piece method [63] at room temperature (25 °C) with the aid of a DCAT 21 (DataPhysics Instruments Gmbh, Filderstadt Germany). All instrument parameters were set as follows: testing length 19.90 mm, width 0.20 mm, motor speed 1.00 mm/s, surface detection threshold 8.00 mg, samples/s 5.00 Hz, immersion depth 3.00 mm.

### 3.6. Measurement of Rheological Behavior of Emulsions

The rheological behavior of emulsions was measured as described by other reports [31]. Experimental flow curves were fitted to the Herschel–Bulkley model, *σ = σ*_0_ *+ κγ^n^*, where *σ* is the shear stress (Pa), *σ*_0_ is the yield stress (Pa), *κ* is the consistency index (Pa·s^n^), *γ* is the shear rate (s^−1^) and n is the flow behavior index (dimensionless).

### 3.7. Evaluation of the Chemical Stability of Emulsions

#### 3.7.1. pH

After being pH-adjusted from 1 to 10, 15 mL emulsions were transferred into small beakers (25 mL), tightly sealed with preservative membrane, and then stored at room temperature without light for 2 h. β-carotene retention was measured after storage.

#### 3.7.2. Thermal Treatment

Proper amounts of emulsions were transferred into small beakers (25 mL), then heated in a water bath for 2 h without light, and the temperature was set to 30 °C, 40 °C, 50 °C, 60 °C, 70 °C, 80 °C, or 90 °C. β-carotene retention was measured after thermal treatment immediately.

#### 3.7.3. UV Radiation

Proper amounts of emulsions were transferred into small beakers (25 mL), and then treated under UV radiation at room temperature with the aid of an Intelli-ray 400 Shuttered UV Floodlight (Uvitron Corp., West Springfield, MA, USA). The lamp type was 400 Watt Metal Halide and its radiation flux was 72 Watts. In this study, the maximum irradiance level was applied. The distance between the bottom of lamp and each shelf was about 5.1 cm, 9.5 cm, 14.0 cm, and 18.4 cm, respectively. The distance between the bottom of lamp and the top surface of each sample was about 18 cm. β-carotene retention was measured after UV radiation treatment for 0, 2, 4, 6, 8, 10 h. The temperature of all emulsions studied did not change significantly after UV radiation.

#### 3.7.4. Strong Oxidant

A 20 mL quantity of emulsion and 40 μL of NaClO were transferred into a small beaker (25 mL), tightly sealed with preservative membrane, and then stored at room temperature without light. β-carotene retention was measured after reaction for 0, 5, 10, 15, 20, 40, 60, 80, 100, 120 min.

#### 3.7.5. Storage Time

Proper amounts of emulsions were transferred into small beakers (25 mL), tightly sealed with preservative membrane, and then stored at 25 °C without light. β-carotene retention was measured after storage for 0, 1, 3, 5, 7, 14, 21, 28, 35 days.

### 3.8. Analysis of β-Carotene Content

The β-carotene content in the emulsions was determined as explained by other researchers [64]. The β-carotene encapsulation rate was defined as C/C_0_, where C is the initial concentration of β-carotene in fresh emulsions and C_0_ is the β-carotene concentration in the emulsion system before first shearing. The β-carotene retention was expressed as C_X_/C, where C_X_ is the concentration of emulsions in any experiment.

### 3.9. Statistical Analysis

All experiments were performed in at least triplicate and the results were expressed as means ± standard deviation (SD) in this study. SD represents the variability of the sample. All data were analyzed using the software SPSS 22.0 (SPSS Inc., Chicago, IL, USA). Each datapoint was measured using an independent random sample which came from a population with normal distribution. Therefore, all data could be analyzed by variance analysis. Statistical differences were determined by one-way analysis of variance (ANOVA) with Duncan’s post hoc test, and differences were considered to be significant when *p* < 0.05. Figures were drawn using the OriginPro 8.5 software (OriginLab Corp., Northampton, MA, USA).

## 4. Conclusions

In comparison with monolayer emulsions (GA-e and BSA-e), the bilayer emulsion BSA/GA-e had more perfect properties, including small size and size distribution, strong potential, high viscosity, good physical stability, high encapsulation ratio of β-carotene, and low interfacial tension. Besides this, the bilayer structure of BSA/GA-e was dense and thick. As a result, BSA/GA-e could weaken the influence of low pH, thermal treatment, UV radiation, oxidants in aqueous phase, and storage time on the chemical stability of β-carotene. On the other hand, the optimal pH for BSA/GA-e was 4, under which most microbes cannot survive. Therefore, BSA/GA-e can be applied in the production of some soft drinks (pH = 2.5 ~ 4) [21]. In addition, the raw materials are widely distributed, cheap, nutritious, biodegradable, and able to meet the requirements of consumers for functional foods, which is conducive to the realization of industrial production. On the other hand, these delivery systems should also effectively protect the load substances in the gastrointestinal system of human body to enhance the biological acceptance of functional substances, in addition to physical and chemical stability in vitro. Therefore, it is an important and difficult problem to design a targeted, controlled, and sustained release delivery system with the gastrointestinal environmental response. Further work needs to be done in the future to study the digestion properties of BSA/GA-e.

## Figures and Tables

**Table 1:** The characteristic parameters of the three emulsions studied.

Emulsion Types	*Z*-Average Diameter (nm)	PDI	Zeta Potential (mV)	β-Carotene Encapsulation Ratio (%)	Interfacial Tension (mN/m)
GA-e	774 ± 22 ^a^	0.43 ± 0.03 ^a^	−31.93 ± 0.67 ^b^	90.1 ± 1.2 ^b^	50.45 ± 0.21 ^a^
BSA-e	199 ± 6 ^c^	0.21 ± 0.01 ^b^	41.47 ± 0.71 ^a^	95.1 ± 1.5 ^a^	41.12 ± 0.77 ^b^
BSA/GA-e	221 ± 5 ^b^	0.23 ± 0.02 ^b^	−30.57 ± 0.71 ^b^	94.4 ± 0.7 ^a^	40.81 ± 0.86 ^b^

PDI, polydispersity index; CI, creaming index. Values are means ± SD (*n* = 3). SD represents the variability of the sample. Different superscript letters in the same column indicate significant differences (*p* < 0.05).

**Table 2:** The rheological parameters from the Herschel–Bulkley model of the three emulsions studied.

Emulsion Types	*σ*_0_ (10^−3^ Pa)	*κ* (10^−3^ Pa·s^n^)	*n*	R^2^
GA-e	7.60 ± 0.41 ^a^	2.70 ± 0.11 ^b^	0.96 ± 0.01 ^a^	0.9995
BSA-e	2.73 ± 0.20 ^c^	2.19 ± 0.06 ^c^	0.92 ± 0.01 ^c^	0.9992
BSA/GA-e	5.31 ± 0.20 ^b^	3.31 ± 0.08 ^a^	0.93 ± 0.01 ^b^	0.9998

Fitted with the Herschel–Bulkley model: *σ* = *σ*_0_ + *κγ^n^*; *σ*, shear stress (Pa); *σ*_0_, yield stress (Pa); *κ*, consistency index (Pa·s^n^); *γ*, shear rate (s^−1^); *n*, flow behavior index (dimensionless). Values are means ± SD (*n* = 3). SD represents the variability of the sample. Different superscript letters in the same column indicate significant differences (*p* < 0.05).
